# Therapeutic cancer vaccines: navigating clinical translation and multimodal synergy

**DOI:** 10.3389/fimmu.2026.1818121

**Published:** 2026-05-12

**Authors:** Tan-Huy Chu, Thuy Linh Huynh, Le-Tri Phuong

**Affiliations:** 1 Immunology Research Division, Tam Anh Research Institute, Ho Chi Minh, Vietnam; 2 Executive Office, Tam Anh Research Institute, Ho Chi Minh, Vietnam

**Keywords:** cancer immunotherapy, epitope spreading, minimal residual disease, therapeutic cancer vaccines, tumor microenvironment

## Abstract

Over the past decades, the field of cancer treatment has been revolutionized by cancer immunotherapy. With therapeutic cancer vaccines (TCVs) are emerging as a promising strategy capable of eliciting potent and lasting T cell responses against cancer cells. While theoretically potent, the translation of TCVs into consistent clinical success remains an evolving challenge. Therefore, this review provides a comprehensive overview of TCVs, traversing from fundamental TCVs concepts, epitope spreading, antigen selection, and delivery platforms, to the current clinical landscape. We specifically examine the transition from monotherapy to innovative combination regimens and propose a translational concept of the strategic utility of TCVs in targeting minimal residual disease (MRD). Despite significant immunogenic potential, the clinical impact of TCVs is currently constrained by the manufacturing hurdles, immunosuppressive tumor microenvironment (TME), patient heterogeneity, and evaluation of outcomes. By addressing these barriers through rational therapeutic approaches and optimized patient selection, TCVs may offer a promising pathway for integration into future clinical paradigms to improve patient outcomes.

## Introduction

In the 21st century, cancer represents a formidable global challenge, impacting human well-being, healthcare systems, and economic stability. A staggering estimate of 20 million new cancer cases were reported globally, accompanied by 9.7 million cancer-related deaths in 2022 (1). Current estimations indicate that approximately one in five individuals will develop cancer during their lifetime, irrespective of sex. Cancer is currently responsible for nearly one in six deaths (16.8%) worldwide and accounts for one in four deaths (22.8%) from noncommunicable diseases, highlighting the urgency of this global challenge (1). Conventional cancer therapies, comprising surgery, radiotherapy, and chemotherapy, are commonly utilized as first-line treatments. However, they are frequently associated with considerable toxicity and constrained applicability. Furthermore, a significant number of cancer patients ultimately develop disease relapse or become refractory to conventional therapies, which necessitates the urgent development of more efficacious therapies for cancer (2–4).

The concept of cancer immunotherapy traces its origins to the late 19^th^ century, driven by the groundbreaking observations of American surgeon William Coley. Coley noticed a phenomenon called spontaneous cancer regression, where the tumors of some patients unexpectedly shrank or even disappeared following a severe bacterial infection. Inspired by the link between infection and anti-tumor activity, Coley began treating patients by injecting a mixture of inactivated bacteria, later called “Coley’s toxins”, directly into tumors. While the treatment was associated with significant side effects, it yielded remarkable results, though inconsistent, with some patients showing positive responses, with tumors shrinking in size (5). Although the underlying mechanism was not fully understood at the time, Coley’s therapy worked by leveraging the potent immune-stimulating properties of the bacterial components, such as pathogen-associated molecular patterns (PAMPs). The Coley’s toxins effectively served as a non-specific immunoadjuvant, triggering an inflammatory response that activated the patient’s macrophages, dendritic cells, and later on T cells to recognize and attack the cancer cells. While his methodology was initially a subject of significant debate, it laid the foundation for the concept of cancer immunotherapy, paving the way for major discoveries later on. In recent years, immunotherapy has emerged as a pivotal strategy in the fight against cancer (6–15). Several cancer immunotherapies, notably immune checkpoint inhibitors, cancer vaccines, oncolytic viruses, oncolytic bacteria, chimeric antigen receptor T (CAR-T) cells, and immune cell engagers have demonstrated encouraging results in both preclinical and clinical studies (2–4, 6, 16–19). Among these diverse approaches, cancer vaccines hold a unique position. Though originally designed to prevent infectious diseases, their ability to activate immune responses is now recognized as a promising therapeutic tool for combating cancer (2, 3, 16, 20, 21). Cancer vaccines are broadly categorized into preemptive cancer vaccines and therapeutic cancer vaccines (TCVs), distinguished by their clinical intent and the immunological status of the host. Preemptive vaccines aim to prevent cancer development by training the immune system to recognize and eliminate oncogenic agents, such as human papillomavirus (HPV) or hepatitis B virus (HBV). On the other hand, TCVs aim to treat existing cancer by stimulating the immune system to specifically recognize and kill cancer cells (3, 22). In this review, we focus on TCVs. We commence by describing their fundamental concept, followed by a critical assessment of the primary barriers to clinical success, including antigen selection and antigen delivery systems. Finally, we discuss the current clinical landscape of TCVs, specifically addressing their therapeutic approaches and evaluation of outcomes.

## Concept of therapeutic cancer vaccines and epitopes spreading

**Figure 1:**
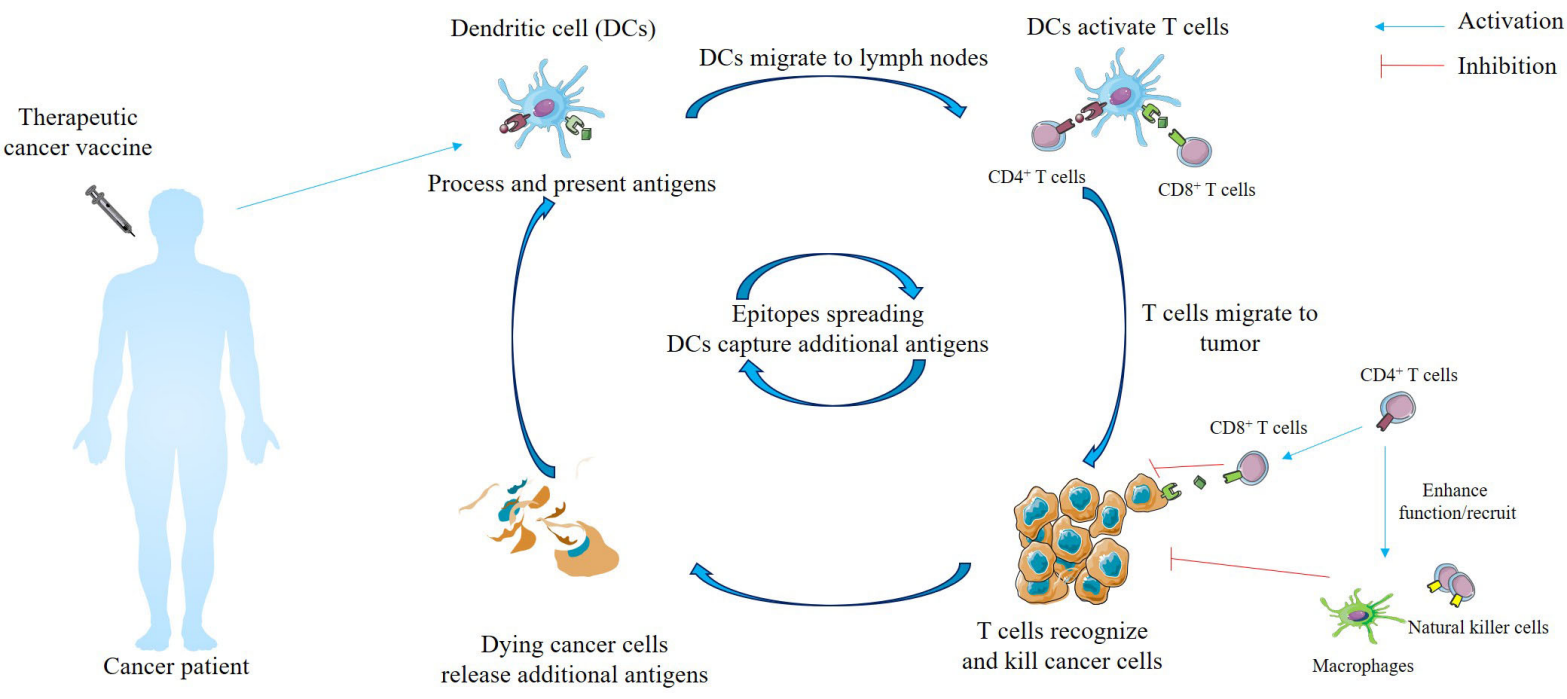
Concept of therapeutic cancer vaccine. Following immunization, antigen-presenting cells (APCs), particularly dendritic cells (DCs), capture and proteolytically process cancer antigens for loading onto major histocompatibility complex (MHC) class I and II complexes. These activated DCs migrate to regional lymph nodes to prime CD4^+^ and CD8^+^ T cells, which subsequently infiltrate the tumor site guided by chemokine gradients. Within the tumor microenvironment (TME), activated T cells execute tumor clearance through direct cytotoxicity, and indirect signaling via IFN-γ and TNF-α. These cytokines polarize macrophages toward a pro-inflammatory M1 phenotype and recruit natural killer (NK) cells to amplify the anti-tumor response. Furthermore, vaccine-induced tumor cell death triggers epitope spreading, a process where the release of secondary antigens diversifies the T cell repertoire and facilitates a broader, more durable immune response.

In the concept of TCVs, after immunization, cancer antigens are captured by antigen-presenting cells (APCs), primarily dendritic cells (DCs). Once internalized by DCs, cancer antigens undergo proteolytic processing and are subsequently loaded onto major histocompatibility complex (MHC) class I and class II complexes. Subsequently, these activated DCs migrate to the lymph nodes, where the MHC I/II complexes bind to and activate CD4^+^ and CD8^+^ T cells (16). The activated T cells then migrate to the tumor site, drawn by chemokine gradients (16). Once infiltrated into the tumor microenvironment (TME), these activated T cells execute tumor eradication through two primary ways. First, they exert direct cytotoxicity via the release of perforin and granzymes, which induce osmotic lysis and DNA fragmentation, and through the Fas/Fas-ligand (FasL) apoptotic pathway. Second, T cells orchestrate an indirect antitumor response through cytokine-mediated signaling, IFN-γ and TNF-α; these signals polarize macrophages toward a pro-inflammatory M1 phenotype and recruit natural killer (NK) cells, collectively amplifying the immune response against the malignant architecture (**Figure 1**) (4, 16). The efficacy of TCVs can be evaluated through a comparative analysis of tumor tissue obtained via core biopsies, both pre and post-treatment. A favorable clinical response has been demonstrably correlated with the post-treatment infiltration of CD3^+^ T cells at the tumor site (3, 23). Furthermore, the dying cancer cells following TCVs treatment lead to the release of additional antigens, a phenomenon termed epitope spreading. This process diversifies the T cell repertoire, consequently eliciting a broader and lasting anti-tumor immune response (**Figure 1**). An influential study involved eight melanoma patients who had received the neoantigen vaccine (NCT01970358), with four years of follow-up post-vaccination. At the time of evaluation, all patients were alive, and six exhibited no evidence of active disease. The vaccine successfully induced durable T cell responses specific to neoantigens; in addition, evidence of epitope spreading was observed, suggesting on-target vaccine-induced tumor cell killing (24). Subsequently, in another important phase Ib clinical trial, thirty-eight patients diagnosed with non-squamous non-small cell lung cancer received treatment consisting of the neoantigen vaccine NEO-PV-01 in combination with pemetrexed, carboplatin, and anti-PD-1 (NCT03380871). The vaccine successfully induced neoantigen-specific T cell responses; furthermore, epitope spreading to other non-vaccinating neoantigens was observed, including responses to KRAS G12C and G12V mutations (25). Thus, epitope spreading plays a crucial role in TCVs; it is essential for overcoming tumor heterogeneity and preventing immune escape, ultimately boosting vaccine effectiveness and potentially extending patients’ quality of life and overall survival (OS).

## Antigen selection in therapeutic cancer vaccines

Selecting optimal tumor antigens for vaccine development presents a significant challenge. In oncology and immunology, tumor antigens are generally classified into two main categories, tumor-associated antigens (TAAs) and tumor-specific antigens (TSAs). This classification is based on their expression patterns and origin, with distinct implications for immunotherapy (3).

TAAs are generally overexpressed in tumor cells, but also present at lower levels in some normal tissues. The clinical trial landscape for TCVs targeting TAAs has seen significant developments in recent years, from the clean bench to clinical trials. An illustrative instance is carcinoembryonic antigen (CEA), which is overexpressed in a wide range of human carcinomas, including colorectal, gastric, pancreatic, non-small cell lung, and breast carcinomas. Encouraged by the promising results observed in animal models, the development and initiation of clinical trials for a CEA-based vaccine have been significantly supported. These subsequent clinical investigations, utilizing CEA-based cancer vaccines, have demonstrated encouraging outcomes in a subset of cancer patients, specifically manifesting as a delay in tumor progression and extended survival (26). BNT111, an mRNA-based cancer vaccine, is engineered to present four distinct TAAs (NY-ESO-1, MAGE-A3, tyrosinase, and TPTE), all of which are highly expressed in melanoma. Data from a phase I study (NCT02410733) involving patients with unresectable Stage IIIB-C and Stage IV melanoma demonstrated that BNT111 possessed a favorable safety profile and consistently induced robust immune responses. Furthermore, recent positive outcomes from its phase II clinical trial (NCT04526899) indicate that the administration of BNT111 in conjunction with anti-PD-1 can significantly improve the overall response rate (ORR) within a challenging cohort of patients afflicted with advanced, treatment-refractory melanoma (27). Another hallmark instance in the field is sipuleucel-T, the first U.S. Food and Drug Administration (FDA)-approved autologous dendritic cell-based vaccine designed to target prostatic acid phosphatase (PAP) antigens (28, 29). Clinical data demonstrated its efficacy in extending patient survival, with a median overall survival of 25.8 months in the treatment arm compared to 21.7 months in the placebo arm, a statistically significant gain of 4.1 months. Furthermore, the long-term clinical benefit was evidenced by a 36-month survival probability of 31.7% for patients receiving sipuleucel-T, notably higher than the 23.0% observed in the control group (28).

TSAs are characterized by their exclusive expression within malignant cells and total absence from healthy somatic tissue. These antigens primarily arise from two distinct biological sources: the integration of oncogenic viral sequences and the generation of neoantigens. This latter category encompasses novel peptide sequences derived from the tumor’s genomic and transcriptomic instability, including non-synonymous somatic mutations and aberrant alternative splicing events that create unique non-self junctional sequences (3, 30). Vaccines targeting TSAs in virally-induced cancers have been a long focus, these strategies prioritize the targeting of shared viral antigens derived from oncogenic infections that are consistently associated with specific cancer types. With recent advancements refining these approaches, often integrated with other therapies. In a recent phase II clinical trial, the vaccine was designed to target Human Papillomavirus 16 (HPV16) in patients with HPV16-positive cervical intraepithelial neoplasia grade 3 (CIN3). Outcomes revealed that nearly all patients experienced lesion regression, with 50% achieving histopathologic complete response (31). In another phase II clinical trial, the GX-188E vaccine to target HPV in patients with HPV16 or HPV18 positive cervical center, in conjunction with anti-PD-1 therapy; outcomes revealed that at 24 weeks, 11 of 26 evaluable patients (42.3%) achieved an overall response, with 4 patients (15.4%) achieved a complete response (32). On the other hand, personalized cancer vaccines have recently attracted significant attention, largely driven by breakthroughs in high-throughput gene sequencing and a more comprehensive understanding of neoantigen production (3). In an important phase II clinical trial (NCT03815058), the personalized mRNA neoantigen vaccine (BNT122) demonstrated promising results for patients with resected pancreatic ductal adenocarcinoma. Administered in combination with anti-PD-L1 and mFOLFIRINOX chemotherapy, the vaccine was found to be safe and induced robust neoantigen-specific T cell responses in approximately half of the treated patients. Crucially, a strong correlation was observed between the induction of this vaccine-induced immune response and a significantly reduced risk of cancer recurrence, with sustained benefits seen at a 3-year follow-up (33).

**Figure 2:**
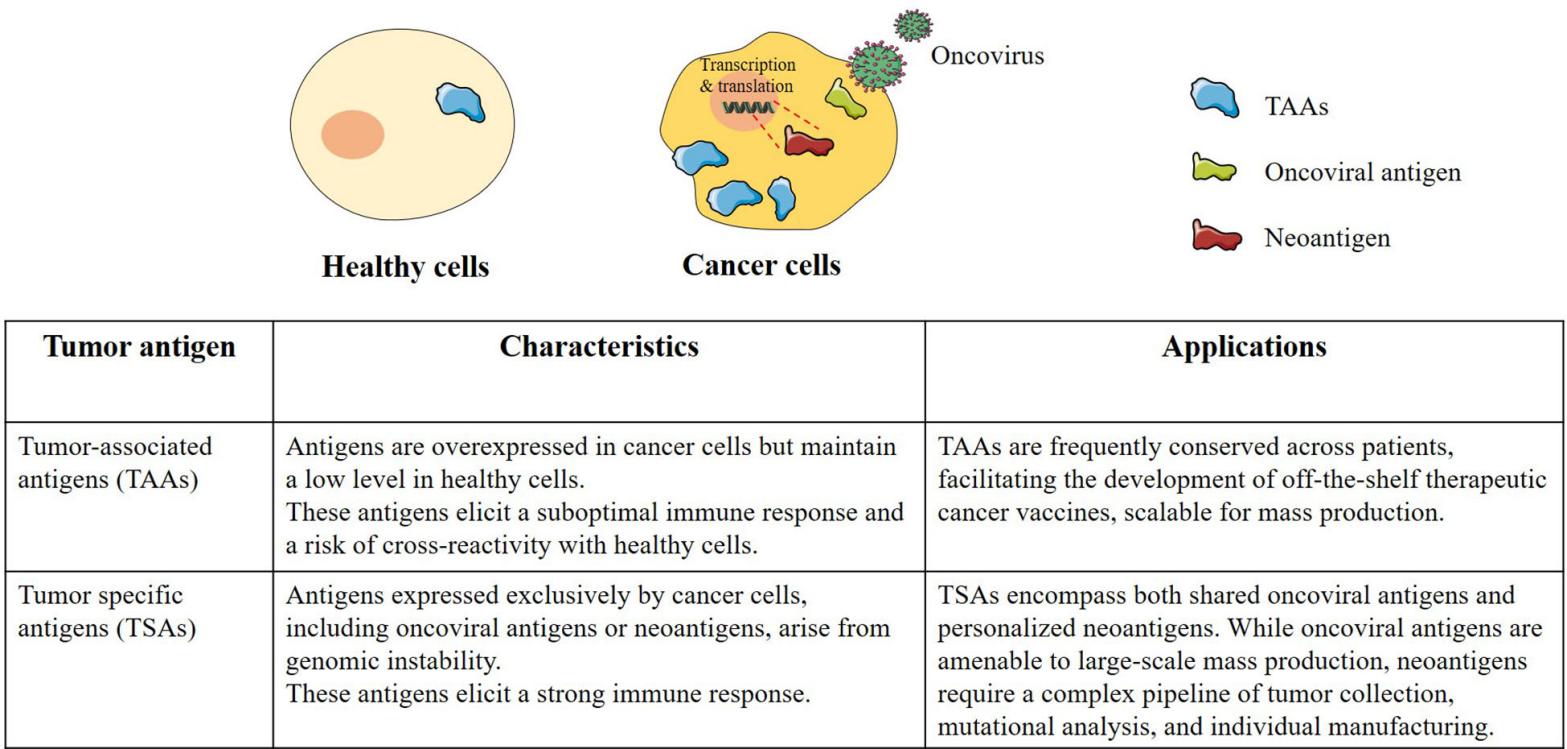
Characteristics and application of tumor antigens in therapeutic cancer vaccine. Tumor-associated antigens (TAAs) are self-proteins overexpressed in malignant cells but present at basal levels in healthy cells, which can lead to suboptimal immune responses and a risk of off-target cross-reactivity. However, their conservation across patient populations makes TAAs ideal for developing off-the-shelf therapeutic cancer vaccines. In contrast, tumor-specific antigens (TSAs), arising from genomic instability (neoantigens) or viral integration (oncoviral antigens), are exclusively expressed by cancer cells, thereby eliciting robust immune responses. While shared oncoviral TSAs facilitate large-scale manufacturing, targeting personalized neoantigens necessitates a complex, highly individualized pipeline encompassing tumor collection, mutational profiling, and bespoke vaccine synthesis.

With TAAs, being overexpressed self-proteins and also present in normal tissues, leading to thymic central tolerance; highly reactive T cells are deleted, and low-affinity ones or regulatory T cells (Tregs) may persist, hindering robust anti-tumor responses. Conversely, TSAs are unique to tumor cells, thus bypassing thymic central tolerance mechanisms. This enables the selection of high-affinity T cells against TSAs, thereby facilitating more potent anti-tumor immune responses with a significantly lower risk of systemic autoimmunity. While TAAs and shared TSAs, such as oncoviral antigens, offer the advantage of off-the-shelf production and reduced costs, the last decade has shifted focus toward personalized neoantigens, an approach that necessitates a highly sophisticated and resource-intensive workflow (**Table 1**). This pipeline begins with the surgical collection of cancer tissue and peripheral blood, followed by comparative whole-genome sequencing (WGS) or whole-exome sequencing (WES) to accurately delineate somatic mutations from the germline sequence. Subsequently, RNA sequencing is employed to verify that these identified mutations are actively transcribed and capable of being translated into neoepitopes (3). To ensure these peptides can be presented to the immune system, precise human leukocyte antigen (HLA) typing and MHC binding prediction algorithms are utilized to calculate the binding affinity of the mutant peptides to the patient’s specific MHC molecules. Finally, the most immunogenic candidates are synthesized using an appropriate delivery platform. Despite their therapeutic precision, neoantigen-based vaccines face substantial hurdles, including the immense burden of cost, the requirement for specialized human expertise, and the logistical complexity of manufacturing a bespoke product for every patient (3) (**Figure 2**).

**Table 1:** Overview of completed therapeutic cancer vaccine clinical trials (2009–present).

Completion year	NCT number	Trial phase	Cancer type	Vaccine Platform/antigen type	Vaccine name + treatment regimen	Number of patients	Results
2009	NCT00065442	III	Metastatic castration-resistant prostate cancer	Cell/TAAs	Sipuleucel-T	512	OS in vaccination vs placebo: 25.8 vs. 21.7 months; immune responses: 66.2%; acceptable safety profile.
2009	NCT00398138	I	Acute myeloid leukemia	Peptide/TAAs	WT-1 analog peptide vaccine	10	WT1-specific T cell response: 7/8 patients (87.5%); acceptable safety profile.
2009	NCT00094653	III	Metastatic melanoma	Peptide/TAAs	Gp100 vaccine + Anti CTLA-4	676	2-year OS (ipilimumab vs ipilimumab + gp100): 25% (24/95) vs 19% (54/284); acceptable safety profile.
2010	NCT00399529	II	Breast Neoplasms	Cell/whole tumor cell antigens	Allogeneic GM-CSF–secreting breast cancer vaccine + Anti-HER2 + Chemotherapy	20	Clinical benefit rates: 55% (6 months), 40% (1 year); immune responses: 35%; acceptable safety profile.
2010	NCT00118274	I/II	Stage IIB to IV melanoma	Peptide/TAAs	MELITAC 12.1 (12MP + tetanus helper) or MELITAC 12.6 (12MP + 6MHP helper) + Chemotherapy	170	Significantly higher CD8^+^ T cell responses were observed with MELITAC 12.1 compared to MELITAC 12.6 (78% vs 19%); cyclophosphamide had no significant effect on T cell responses; one treatment-related grade 4 toxicity (hypoglycemia).
2011	NCT00616941	I	Advanced ovarian cancer	Peptide/TAAs	NY-ESO-1 OLP4	28	NY-ESO-1–specific CD8^+^ T cell response: 64%; CD4^+^ T cell response: 100%; acceptable safety profile.
2012	NCT00952692	I/II	HER2-overexpressing breast cancer	Protein/TAAs	dHER2 + Anti-HER2	12	HER2-specific T cell response: 8%; acceptable safety profile.
2012	NCT00836407	I	Advanced pancreatic ductal adenocarcinoma	Cell/whole tumor cell antigens	GVAX + Anti-CTLA-4	30	Median OS in anti-CTLA-4 vs combination of anti-CTLA-4 and GVAX: 3.6 vs. 5.7 months (HR: 0.51, P = 0.072); grade 3–4 IRAEs: 20% in both arms.
2013	NCT01989572	III	High-risk melanoma	Peptide/TAAs	Multiepitope vaccine +/- GM-CSF	815	No significant OS or RFS benefit; acceptable safety profile.
2014	NCT01431391	II	Biochemically recurrent prostate cancer	Cell/TAAs	Sipuleucel-T administered before or after ADT	68	PA2024-specific T cell responses were similar in both arms (sipuleucel-T before ADT vs sipuleucel-T after ADT: 29/33 (88%) vs 29/34 (85%) (P = 1.00); acceptable safety profile.
2016	NCT01266083	II	Acute Myeloid Leukemia	Peptide/TAAs	Galinpepimut-S	22	Immune responses: 64% (9/14); acceptable safety profile.
2016	NCT01487863	II	Metastatic castration-resistant Prostate Cancer	Cell/TAAs	Sipuleucel-T + Hormone therapy	69	Median OS: 33.3 months; acceptable safety profile.
2017	NCT01147965	I/II	Metastatic colorectal cancer	Viral vector/TAAs	Ad5 CEA	32	CEA-specific T cell responses: 61% (19/31); 12-month OS: 48%; acceptable safety profile.
2017	NCT00450463	II	Non-metastatic castration resistant prostate cancer	Cell/TAAs	Sipuleucell-T + Anti-androgen	64	Longer time to treatment failure vs anti-androgen alone; acceptable safety profile.
2017	NCT00490529	II	Mantle cell lymphoma	Cell (CpG-activated)/whole tumor cell antigens	CpG-MCL Vaccine	45	High MRD-negative rate (89%); vaccine-induced CD8^+^ T cell responses in 40% associated with favorable outcomes; acceptable safety profile.
2018	NCT02179515	I	Advanced cancers	Viral vector/TAAs	MVA-brachyury- TRICOM	38	Brachyury-specific T cell responses: 82% (28/34); acceptable safety profile.
2018	NCT02981524	II	Advanced mismatch repair proficient colorectal cancer	Cell/whole tumor cell lysates	(GVAX + Chemotherapy) + Anti-PD-1	17	Median OS: 213 days; ≥30% CEA decline: 41%; grade ≥3 TRAEs in 2/17 patients.
2018	NCT02129075	II	Malignant Melanoma	Protein/TAAs	CDX-1401 + Growth factor	60	NY-ESO-1–specific T cell responses: 100% (30/30) with combination vs 73% (22/30) with vaccine alone; acceptable safety profile.
2018	NCT01570036	II	Breast Cancer	Peptide/TAAs	Nelipepimut-S + Anti-HER2	275	No significant difference in DFS between combination and anti-HER2 therapy alone; acceptable safety profile.
2018	NCT01266460	II	Advanced cervical cancer	Bacterial vector/ viral antigen	ADXS-HPV	50	12-month OS: 38%; median OS: 6.1 months; acceptable safety profile.
2019	NCT01433172	I/II	Advanced lung adenocarcinoma	Cell/whole tumor cell lysates	GM.CD40L.CCL21	65	6-month PFS: 15.2% (GM.CD40L) vs 9.4% (GM.CD40L.CCL21); acceptable safety profile.
2019	NCT03199872	I/II	Prostate Cancer	Peptide/TAAs	RV001V	22	CD4+ T cell response observed: 86% (18/21); acceptable safety profile.
2019	NCT03391232	I/II	Metastatic colorectal cancer	Peptide/TAAs	PolyPEPI1018	11	CD8+ T cell responses: 80%; acceptable safety profile.
2019	NCT01675765	I	Malignant Pleural Mesothelioma	Bacterial vector/TAAs	CRS-207 + Chemotherapy	35	Clinical response: 57%; acceptable safety profile.
2021	NCT03481816	I	Metastatic castration-resistant prostate cancer	Viral vector/TAAs	ETBX-071/061/051	18	T cell response: 100%; acceptable safety profile.
2021	NCT02126579	I/II	Stage IIB-IV melanoma	Peptide/TAAs	LPV7	50	CD8^+^ T cell response: 18%; overall T cell response: 30%; acceptable safety profile.
2022	NCT03689192	I	Refractory solid tumors	Peptide/TAAs	ARG1-18,19,20	10	T cell response: 90%; acceptable safety profile.
2022	NCT03047928	I/II	Metastatic Melanoma	Peptide/TAAs	IO102/IO103 + Anti-PD-1	30	Clinical response: 80%, including 43% CR; T cell response: 93%; acceptable safety profile.
2022	NCT02301611	II	Resected stage III/IV melanoma	Cell/ Tumor cell lysates	TLPLDC (± G-CSF) or TLPO	187	TLPLDC (without G-CSF) and TLPO significantly improved DFS and OS compared with placebo (DFS up to 60.9% vs 27.2%; OS up to 94.6% vs 62.5%); two related adverse event ≥grade 3.
2023	NCT00194714	I/II	Stage IV HER2/neu-positive breast cancer	Peptide/TAAs	HER-2/neu + Anti-HER2	21	T cell responses: 90%; acceptable safety profile.
2023	NCT02960230	I/II	Diffuse Intrinsic Pontine Glioma	Peptide/ TSAs	K27M peptide	29	CD8^+^ T cell response: 39% (7/18); 12-month OS: ~40%; acceptable safety profile.
2024	NCT02795988	I/II	Gastrointestinal Adeno-carcinoma	Peptide/TAAs	IMU-131 + Chemotherapy	36	Improved OS vs chemotherapy alone (13.9 vs 8.3 months; HR 0.60); acceptable safety profile.

Clinical responses include complete responses (CR) and partial responses (PR). Clinical benefit includes complete responses (CR), partial responses (PR), and stable disease (SD). TAAs, tumor-associated antigens; TSAs, tumor-specific antigens; OS, overall survival; DFS, disease-free survival; CR, complete response; PR, partial response; SD, stable disease; CEA, carcinoembryonic antigen; TRAEs, treatment-related adverse events; HR, hazard ratio; ADT, androgen deprivation therapy; MRD, minimal residual disease.

## Therapeutic cancer vaccine platform

**Figure 3:**
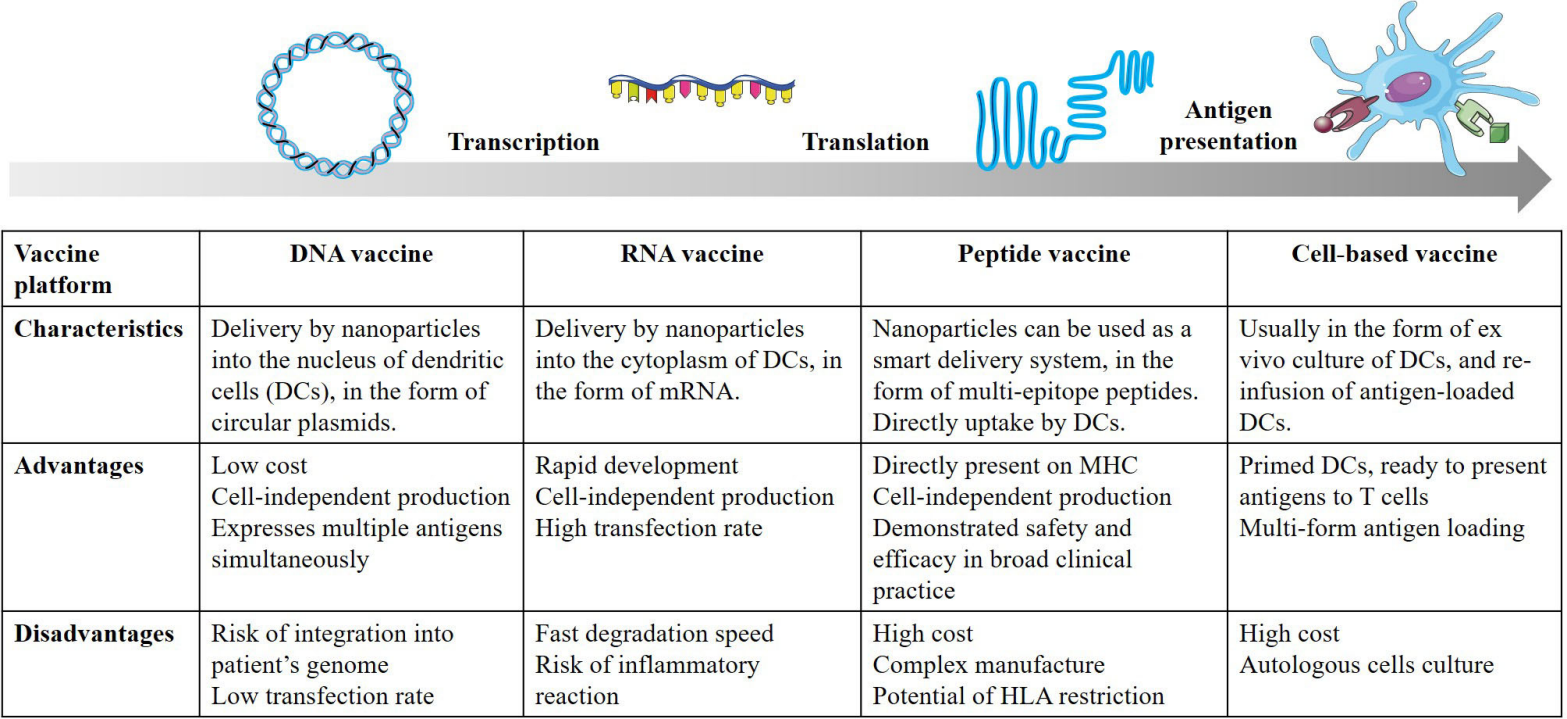
Characteristics, advantages and disadvantages of traditional therapeutic cancer vaccine platform. DNA vaccines, delivered as circular plasmids into the dendritic cells (DCs) nucleus via nanoparticles, offer low-cost, cell-free production of multiple antigens but are limited by low transfection rates and potential genomic integration risks. Conversely, mRNA vaccines are delivered into the DCs cytoplasm, enabling rapid development and high transfection efficiency, though hindered by fast degradation and potential inflammatory reactivity. Peptide vaccines utilize multi-epitope sequences, either taken up directly by DCs or via smart nanocarriers, allowing for direct MHC presentation and proven clinical safety, but are restricted by complex manufacturing, high costs, and human leukocyte antigen (HLA) dependency. Finally, cell-based vaccines rely on the ex vivo culture and re-infusion of autologous, antigen-loaded DCs; while providing optimally primed cells, this approach is significantly constrained by high costs and highly individualized manufacturing pipelines.

Another critical bottleneck in the successful development of TCVs involves the precise delivery of antigens to target APCs to elicit a sustained immune response. Consequently, the selection of an optimal vaccine platform requires a multifaceted evaluation of the platform’s biochemical composition, inherent immunogenicity, and specific delivery kinetics. Utilizing traditional taxonomies, these platforms are categorized according to their foundational technological modalities and the specific biological vehicles employed for antigen delivery, involving DNA-based platforms that utilize plasmid vectors to ensure stable antigen expression along with other immune-stimulatory cytokines (34, 35); mRNA platforms leverage lipid nanoparticle delivery for rapid translation without genomic integration, this platform was recently brought back into the spotlight with the FDA’s emergency approval of two mRNA COVID-19 vaccines (3); peptide-based platforms offer high specificity through synthesized amino acid sequences along with an immune-adjuvant to improve antigen presentation; and cell-based platforms, such as autologous dendritic cells (DCs) primed with multiple antigenic epitopes, which elicit a diverse repertoire of both CD4^+^ and CD8^+^ T cell responses (16, 20, 21). The critical appraisal of the distinct advantages and inherent limitations of these diverse platforms (**Figure 3**), with key clinical trial outcomes summarized (**Table 1**), provides a comparative overview of their translation to clinical efficacy.

Furthermore, the field is rapidly evolving toward next-generation modalities, including viral and bacterial vector vaccines, which represent sophisticated platforms for the targeted delivery of cancer antigens, though their clinical utility often relies on synergistic combinations with conventional cancer therapies. Recombinant viruses function by infiltrating immune cells to deliver high antigenic loads, activate T cell and induce robust anti-tumor responses (36); however, while preclinical results were promising, clinical trials using viral vectors as monotherapies have largely been disappointing. Nevertheless, clinical outcomes improve significantly when paired with other treatments. As evidenced by the clinical trial phase II (NCT02779855), the oncolytic virus T-VEC combined with neoadjuvant chemotherapy, achieved a 45.9% complete pathologic response in triple-negative breast cancer patients, 89% remained disease-free 2 years post-treatment; immune activation during treatment also correlated with response (37). In a similar clinical setting, the clinical trial phase II (NCT00179309), the PANVAC utilizes a poxvirus vector to deliver CEA and MUC1 antigens. When administered in combination with docetaxel, the vaccine has been shown to enhance clinical outcomes for patients with metastatic breast cancer, the combination therapy prolonged the progression-free survival (PFS) to 7.9 months compared to only 3.9 months in the group receiving docetaxel monotherapy (38). These studies underscore the potential of viral vectors to overcome the limitations of monotherapy by augmenting the efficacy of cytotoxic agents through the induction of a robust, antigen-specific immune response. On the other hand, bacteria serve as effective delivery vehicles by exploiting the unique physiology of the TME; strains such as *Bacillus* thrive in anaerobic, hypoxic conditions, while auxotrophic *Salmonella* are attracted to the TME for nutrient uptake (6, 39–41). In a preclinical study, engineered *Salmonella typhimurium* provides a tumor-specific delivery system for a dual payload of cytolysin A and flagellin B. This targeted platform triggered immunogenic cell death and successfully reprogrammed the TME, activating both innate and adaptive immune responses to inhibit metastasis and establish long-lasting antitumor memory without significant systemic toxicity (6, 41). These inherent bacterial traits, including the ability to migrate into remote, poorly vascularized tumor regions, make them promising candidates for the delivery of anti-cancer agents.

Advanced nano-drug delivery systems, such as lipid nanoparticles (LNPs), exosomes, bacterial outer membrane vesicles, and amphiphilic vaccines, are being developed to improve tumor antigen targeting and DCs uptake. Notable innovations include personalized DC-mimicking nanovaccines that utilize Escherichia coli and tumor components, to activate the stimulator of interferon genes (STING) pathway, fostering DC maturation and significant lymph node homing to elicit potent T cell responses (42). Additionally, bilayer lipopolyplex vectors are being studied to deliver mRNA with promising preclinical results, activating toll-like receptor 7/8 signaling and enhancing DCs antigen presentation; this core-shell mRNA vaccine achieved a 90% reduction in metastatic tumor nodules *in vivo* (43). By optimizing antigen delivery to the lymph nodes, these platforms maximize DCs’ antigen uptake and presentation, thereby priming potent T cell activation to improve clinical outcomes.

## Clinical landscape of therapeutic cancer vaccines

### Clinical trial landscape

Over the past few decades, a significantly improved understanding of how cancer cells evade immune system detection has driven remarkable progress in cancer immunotherapy. For instance, immune checkpoint inhibitors (ICIs) and adoptive cell therapies have demonstrated their ability to induce tumor regression in patients with hematological or solid malignancies. These advancements clearly demonstrate the viability of cancer immunotherapy, which in turn has led to a rapid growth in cancer vaccine development, employing strategies that mimic natural immunity.

In this review, we selected clinical trials of TCVs completed since 2009 to the present, a period defined by the emergence of the checkpoint inhibitor era and following the landmark 2009 Phase III clinical trial of Sipuleucel-T (28), one of the first TCVs to receive approval from the U.S. FDA (44). Eligible studies must have comprehensive information on the study timelines, methodology, and results clearly published on both ClinicalTrials.gov and PubMed. The study selection process, including search strategy and inclusion/exclusion criteria, is described in the **Supplementary Appendix** and **Supplementary Figure 1**. Ultimately, thirty-two trials met the inclusion criteria and are summarized in **Table 1**.

The included trials targeted both solid tumors and hematological malignancies, while melanoma has historically been the cornerstone of immunotherapy research, the clinical landscape of TCVs is undergoing a significant strategic expansion. The trials reviewed here indicate increasing investigation across multiple tumor types, including prostate cancer (28, 45–49), melanoma (24, 47, 50–53), breast cancer (54–57), and colorectal cancer (58–60). Fifty-six percent (18/32) of trials in **Table 1** were conducted as Phase I or Phase I/II trials, with fewer Phase II and Phase III trials completed during the study period. This design emphasis highlights the exploratory nature of therapeutic cancer vaccine development over the past seventeen years (61). **Table 1** demonstrates a variety of vaccine platforms, with peptide-based vaccines being the most frequently employed (47%, 15/32), followed by cell-based, protein-based, viral vector–based, and bacterial vector–based approaches. The prevalence of peptide-based vaccines may be attributed to their favorable safety and efficacy profile in clinical practices, alongside the ability to precisely target well-characterized TAAs (62). Notably, DNA- and RNA-based vaccine platforms are not represented among the trials summarized in **Table 1**. This is due to the inclusion criteria of the present review, which required completed clinical trials with publicly available results in ClinicalTrials.gov. Several trials investigating DNA- and mRNA-based cancer vaccines were not included due to the lack of reported results or their ongoing status, including DNA-based vaccine trials in metastatic prostate cancer (NCT02411786) and hepatocellular carcinoma (NCT04251117), as well as an mRNA-based vaccine trial in advanced melanoma (NCT02410733). Collectively, these studies illustrate the diversity of vaccine platforms and design strategies employed in therapeutic cancer vaccine research, spanning both traditional approaches and emerging technologies, and reflecting the ongoing evolution of the field toward more precise and adaptable immunotherapeutic strategies.

Regarding antigen selection, TAAs account for most of the studies (24/32). In contrast, relatively few trials evaluated alternative antigen sources, including whole tumor cell–based vaccines, tumor lysates, TSAs, and viral antigens. Whole tumor cell and lysate-based vaccines provide a broader repertoire of antigens, potentially enabling more comprehensive immune activation and reducing the risk of immune escape due to antigen loss. However, these approaches may be limited by variability in antigen composition, manufacturing complexity, and challenges in standardization. Although only one TSA-based trial is represented in **Table 1**, due to the inclusion criteria of the present review, this still reflects the emergence of neoantigen-based vaccine strategies. This transition likely reflects advances in next-generation sequencing and personalized immunotherapy strategies, enabling more precise targeting of patient-specific mutations (63).

In addition, clinical development of TCVs has progressed from evaluating their efficacy as monotherapy interventions toward integrating them into combination regimens (64). As understanding of tumor immune resistance and the immunosuppressive tumor microenvironment advanced (65), subsequent trials increasingly incorporated TCVs with chemotherapy (24, 54, 59, 66), targeted therapy (56), or immune checkpoint inhibitors (ICIs) (52, 53, 59, 67, 68). In particular, the combination of TCVs and ICIs has gained increasing attention due to their complementary mechanisms of action. While TCVs are designed to prime and expand tumor-specific T cells through enhanced antigen presentation (3), ICIs function by releasing inhibitory brakes on activated T cells, thereby restoring or amplifying antitumor immune responses (16, 21, 69). Consequently, the combination of TCVs with ICIs represents a biologically rational strategy aimed at overcoming tumor immune resistance, thereby contributing to improved clinical outcomes.

Immunological endpoints were assessed in several trials included in **Table 1**. Vaccine-induced immune responses were primarily evaluated using the Enzyme-Linked Immunospot (ELISpot) assay, focusing on antigen-specific T cell activation (70). Several studies demonstrated robust CD4^+^ and CD8^+^ T cell responses, with immune response rates exceeding 70–80% among evaluable patients (51, 71, 72). Despite the frequent induction of antigen-specific immunity, the association between immune activation and clinical benefit remained inconsistent, highlighting the multifactorial nature of tumor control and the challenges in translating immunogenicity into meaningful clinical benefit. In terms of clinical outcomes, particular emphasis was placed on clinical responses, specifically complete response (CR) and partial response (PR), to provide direct and clinically meaningful indicators of antitumor efficacy. For instance, the peptide-based vaccine IO102/IO103 in combination with anti-PD-1 therapy demonstrated a high overall response rate of 80%, including 43% CR, in patients with metastatic melanoma (53). In another study, the bacterial vector vaccine CRS-207 combined with chemotherapy achieved a 57% clinical response rate in patients with mesothelioma, supporting the potential synergy between conventional therapies and immunotherapy (66). In addition, improvements in survival outcomes were observed in selected studies, such as the IMU-131 peptide vaccine combined with chemotherapy, which showed prolonged OS compared to chemotherapy alone (73). However, despite these encouraging findings, clinical efficacy across trials remains variable, and meaningful responses are not consistently observed. This variability may reflect differences in tumor types, disease stages, vaccine platforms, and treatment regimens. Therefore, while objective responses provide valuable indicators of antitumor activity, their interpretation should be approached with caution in the context of heterogeneous study designs.

Collectively, the available data indicate that TCVs may hold greater therapeutic potential when integrated into multimodal immunotherapeutic approaches rather than used as standalone interventions. Furthermore, most of the TCVs trials in **Table 1** demonstrated favorable safety profiles. This consistent tolerability distinguishes cancer vaccines from other systemic therapies and supports their potential use in adjuvant or maintenance treatment settings, where long-term safety is essential (74).

To complement the clinical trials summarized in **Table 1** and to provide a perspective on recent developments not captured within the predefined inclusion criteria, recent studies have further expanded the therapeutic cancer vaccine landscape, highlighting both emerging opportunities and ongoing challenges. For example, the combination of the PROSTVAC vaccine with nivolumab was associated with increased intratumoral T cell infiltration and expansion of antigen-specific T cell responses in patients with prostate cancer, suggesting enhanced immune activation with combined approaches (75). Similarly, a plasmacytoid dendritic cell–based vaccine (PDC*lung01) demonstrated encouraging clinical activity when combined with anti–PD-1 therapy in non-small-cell lung cancer, with objective responses and progression-free survival correlating with vaccine-induced immune responses (76). However, not all combination approaches have translated into improved clinical outcomes. In a randomized phase II trial, the addition of the telomerase-targeted vaccine UV1 to dual ICIs with ipilimumab and nivolumab did not improve progression-free survival or response rates compared with checkpoint inhibition alone (77). Collectively, these recent findings underscore the rapid evolution of TCVs, while also emphasizing the need for improved strategies to translate immunogenicity into consistent clinical benefit.

In summary, the completed clinical trials since 2009 demonstrate that TCVs can reliably induce antigen-specific immune responses with an acceptable safety profile (74). However, limited and variable clinical efficacy highlights the need for effectively targeting the immunosuppressive tumor microenvironment, optimizing therapeutic approaches, and employing more rational frameworks for evaluating outcomes, which is addressed in subsequent parts.

### Targeting the tumor microenvironment

The TME is a complex system that surrounds and protects tumors from immune cells through various immunosuppressive factors, including regulatory T cells (Tregs), M2 macrophages, myeloid-derived suppressor cells (MDSC), cancer-associated fibroblasts, and the presence of programmed cell death-ligand 1 (PD-L1) on the surface of cancer cells. Cells within the TME secrete a potent mixture of immunosuppressive signals, such as transforming growth factor-beta (TGF-β) or vascular endothelial growth factor (VEGF), while simultaneously engaging the PD-1/PD-L1 axis through direct cell-to-cell contact. These mechanisms act synergistically to inhibit effector T cells, effectively neutralizing the host’s anti-tumor immunity (78, 79). For instance, Tregs block T cell activation, M2 macrophages promote tumor growth by inducing angiogenesis, PD-L1 on the surface of cancer cells or DCs suppresses T cell function, cancer-associated fibroblasts secrete a complex extracellular matrix that physically restricts T cells, NK cells, and macrophages infiltration (16, 21, 78)

**Figure 4:**
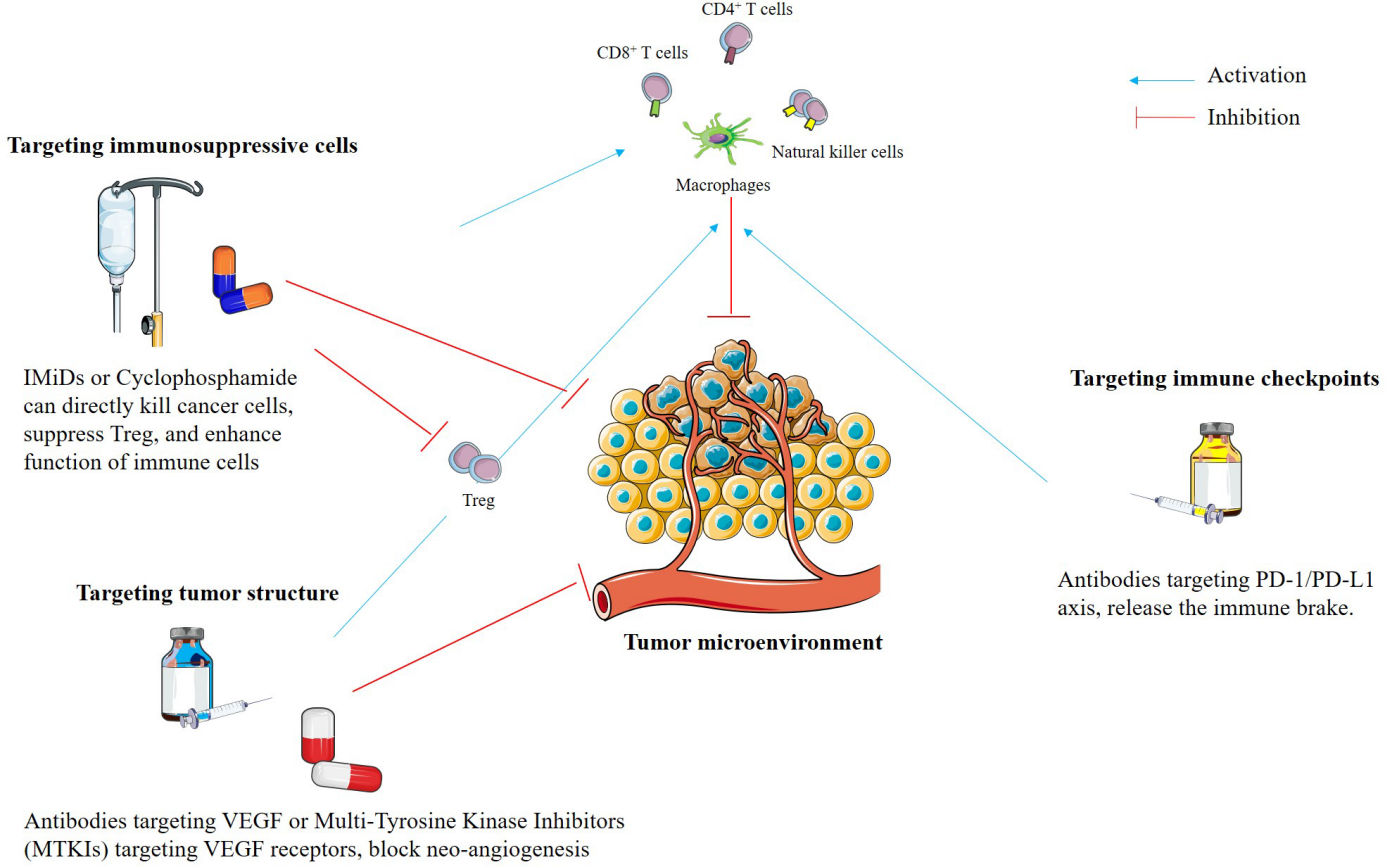
Strategies for modulating the immunosuppressive tumor microenvironment to enhance therapeutic cancer vaccine efficacy. Therapeutic interventions focus on three strategic pillars: immunosuppressive cell depletion, checkpoint blockade, and structural normalization. Depletion of immunosuppressive cells, for instance, such as regulatory T cells (Tregs) can be achieved through low-dose metronomic cyclophosphamide or immunomodulatory imide drugs (IMiDs) like lenalidomide or pomalidomide, which simultaneously augment T cell and natural killer (NK) cell activity. To target the upregulation of inhibitory molecules on vaccine-induced T cells, for instance, inhibition of the PD-1/PD-L1 axis is employed to release the immune brakes and restore cytotoxic potential. Finally, targeting tumor vascular architecture, for example, via anti-VEGF antibodies or multi-tyrosine kinase inhibitors (TKIs) facilitates effective T cell infiltration by normalizing disorganized neo-angiogenesis. Collectively, these combinatorial approaches neutralize tumor microenvironment (TME) mediated resistance, fostering a permissive landscape that maximizes the clinical potency of therapeutic cancer vaccines.

Ongoing research continues to elucidate the intricate complexity and heterogeneity of the TME and its profound influence on therapeutic outcomes. Consequently, neutralizing the immunosuppressive landscape within the TME is a critical strategy for potentiating the clinical efficacy of TCVs. To effectively reverse the suppressive TME, therapeutic strategies are generally categorized into three perspectives: the depletion of immunosuppressive cells, the administration of immune checkpoint inhibitors, and the structural targeting of the tumor to facilitate immune infiltration. By integrating these methods, clinicians can transform the TME from an immunologically cold sanctuary into a hot environment, thereby maximizing the potential of TCVs. For targeting the immunosuppressive cells, to give an instance, low continuous doses of cyclophosphamide target and eliminate Tregs (80). Furthermore, the use of immunomodulatory imide drugs (IMiDs), including thalidomide, lenalidomide, and pomalidomide can inhibit Tregs, while promoting T cells and NK cells function (16, 20, 21, 81). For targeting the immune checkpoints, it is critical to address the fact that antigen-specific T cells generated by TCVs frequently upregulate suppressor molecules. The PD-1/PD-L1 axis is a primary resistance mechanism, providing inhibitory signaling upon T cell receptor recognition that allows cancer cells to evade immunity (16). Consequently, blocking this pathway effectively releases the immune brakes, reversing TME-mediated suppression and restoring the cytotoxic potential of vaccine-induced T cells (3, 16, 21, 82). For targeting the tumor structure, for example, inhibiting angiogenesis through antibodies targeting vascular endothelial growth factor (VEGF) or multi-tyrosine kinase receptor (MTKR) can both block neo-angiogenesis and normalize the tumor vasculature. Consequently, activated T cells can effectively infiltrate the TME (9, 83). Neutralizing these critical immunosuppressive factors within the TME fosters a more permissive environment for anti-tumor immunity, thereby maximizing the therapeutic potency of TCVs. **Figure 4** summarizes the three core components of the immunosuppressive TME and the corresponding therapeutic strategies employed to reverse them.

### Therapeutic approaches and optimal patient selection

Currently, cancer vaccine research is experiencing an intense surge in early-stage clinical investigations worldwide. Previous research has explored predefined TAAs as targets for TCVs. While these shared antigens facilitate mass production, lower costs, and rapid development, their clinical efficacy has been hampered by central immune tolerance, which often limits the potency of the resulting T cell response. In contrast, personalized cancer vaccines, which target neoantigens, have attracted substantial interest in recent years. However, their widespread application is currently limited by several challenges. The primary limitations for personalized cancer vaccines involve the laborious task of neoantigen identification and the complexities of manufacturing these vaccines quickly and cost-effectively. Furthermore, the clinical efficacy of TCVs targeting TAAs or TSAs has consistently been limited when administered as a standalone monotherapy (**Table 1**).

**Figure 5:**
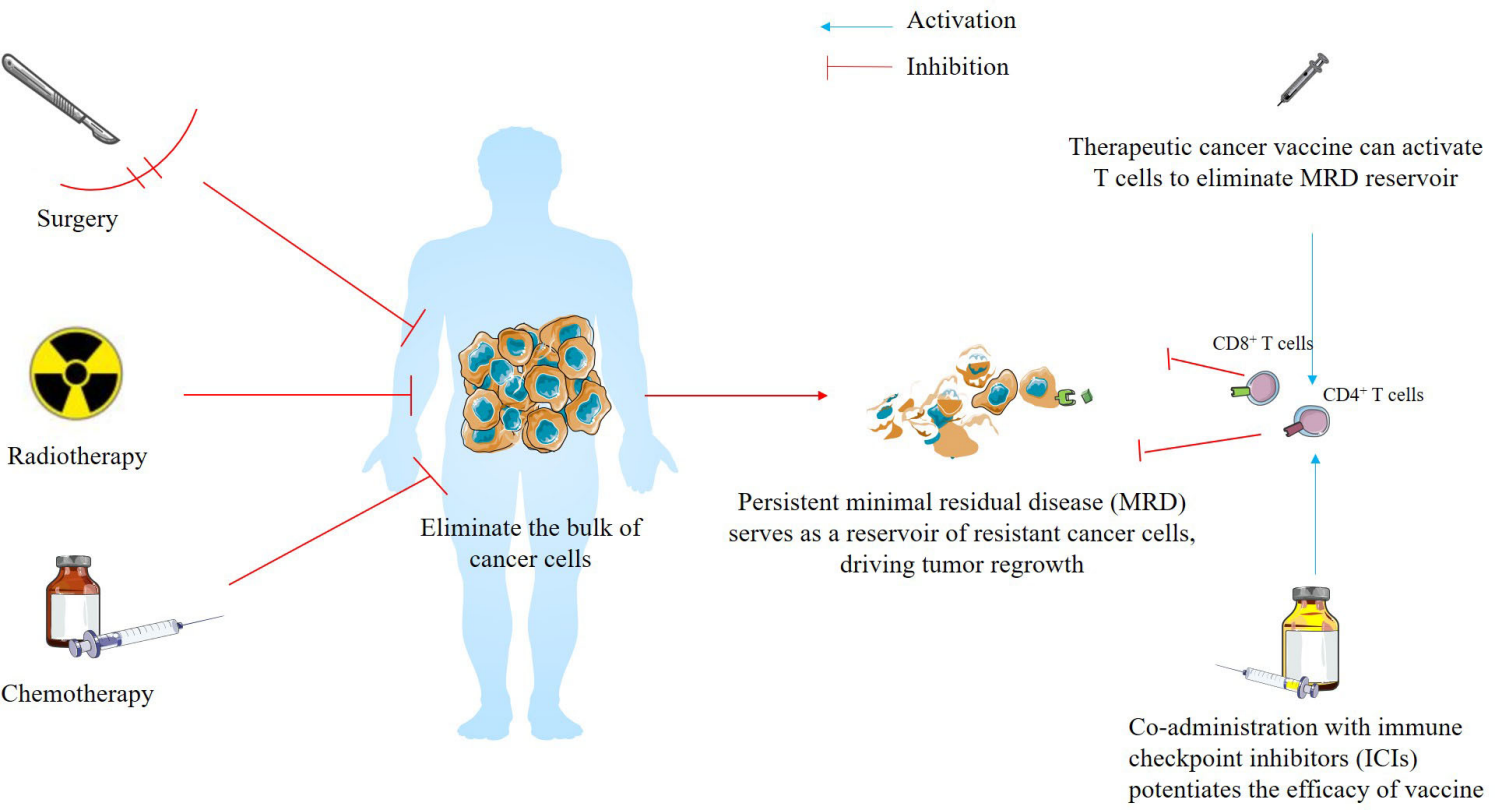
A proposed translational concept for optimizing therapeutic cancer vaccine efficacy: A two-step framework. This model outlines a sequential therapeutic approach designed to optimize anti-tumor immunity. Step I focuses on eliminating the bulk of cancer cells through conventional therapies, including surgery, chemotherapy, or radiotherapy, to minimize primary tumor burden and systemic malignancy. This prepares the physiological landscape for Step II, where therapeutic cancer vaccines (TCVs) are administered during the window of minimal residual disease (MRD). In this setting of low tumor volume, TCVs can more effectively prime antigen-specific T cells to eradicate persistent cancer cells that typically evade conventional therapies. This approach leverages the vaccine’s capacity to establish long-term immunological memory, providing a durable surveillance mechanism to mitigate the risk of recurrence and improve long-term clinical outcomes. In addition, co-administration with immune checkpoint inhibitors (ICIs) can release the immune brake and enhance the function of activated T cells.

In the modern era of clinical practice, a multidisciplinary approach is essential, particularly for managing the intricacies of complex clinical cases (84–90). There is growing interest in developing rational combination regimens featuring a vaccine backbone that targets distinct steps of anti-tumor immunity (2, 16, 91). This highlights a potential synergistic effect when TCVs are combined with other conventional therapies, such as chemotherapy, radiotherapy, or immune checkpoint inhibitors, particularly in cases where these agents might not be efficacious on their own (3). Drawing insights from recent clinical trials data, we propose a two-step therapeutic framework to maximize the efficacy of TCVs. In the initial phase, conventional therapies such as surgery, chemotherapy, or radiotherapy are employed to drastically reduce the primary tumor burden and systemic cancer cell count, with the strategic objective of achieving a minimal residual disease (MRD) positive status. This sets the stage for the second phase, where TCVs are administered within the window of MRD; at this stage, the vaccine can more effectively stimulate antigen-specific T cell populations to eradicate remaining persistent MRD that often evade conventional therapies. Furthermore, this sequential approach leverages the vaccine’s ability to establish robust immunological memory, providing a long-term surveillance mechanism that significantly reduces the risk of cancer recurrence and improves overall clinical outcomes (91–93) (**Figure 5**). Supporting our proposed framework, the landmark Phase I trial of the neoantigen mRNA-4157 vaccine, administered either as monotherapy or in combination with pembrolizumab, demonstrated a manageable safety profile and the induction of potent antigen-specific T cell responses in patients with resected non-small cell lung cancer (n=4) or resected cutaneous melanoma (n=12) (NCT03313778) (94). These findings laid the foundation for the subsequent Phase IIb trial, which evaluated mRNA-4157 plus pembrolizumab (n=107) versus pembrolizumab monotherapy (n=50) in patients with completely resected high-risk cutaneous melanoma (NCT03897881). The combination therapy significantly extended recurrence-free survival (RFS), reducing the risk of recurrence or death compared to monotherapy. Notably, the recurrence/death rate was nearly halved in the combination arm (22% vs. 40%), with an 18-month RFS of 79% compared to 62% for those receiving pembrolizumab alone (95).

In light of these considerations, it is important to emphasize that the two-step therapeutic framework proposed herein represents a translational concept rather than a broadly established clinical strategy. This model synthesizes contemporary immunological rationales with emerging trends from recent clinical trials. While the biological plausibility is robust, we acknowledge that high-level clinical evidence is currently consolidating. Therefore, this framework should be viewed as a conceptual model intended to guide future prospective research and clinical trial designs.

Current cancer vaccine development primarily targets patients with advanced tumors. However, those who have undergone multiple prior lines of therapy often suffer from bone marrow damage and severe immune suppression, hindering vaccine efficacy (3, 23, 88, 89). Shifting vaccines from later-line to first-line treatment could mitigate these risks and improve patient outcomes. Additionally, the typical one to two-month manufacturing period for personalized vaccines is often too long for those with rapidly progressing advanced disease. Focusing on early-stage patients, using TCVs as a neoadjuvant beside conventional therapies, offers a more favorable therapeutic window. In these cases, lower tumor loads or even within the window of MRD, and better physical health may maximize the vaccine’s ability to trigger an immune response before tolerance is established.

### Evaluation of outcomes

Evaluating TCV response accurately requires a shift in focus beyond traditional endpoints like progression-free survival (PFS) or overall survival (OS). Unlike cytotoxic drugs that induce immediate tumor regression through direct tumor killing, vaccines work by inducing a delayed and lasting immune response. This fundamental difference means that conventional measures of tumor regression often fall short in capturing the vaccine’s true and long-term therapeutic impact. Evaluating TCVs efficacy necessitates a comparative analysis of paired tumor biopsies obtained pre- and post-treatment. Such longitudinal immune profiling enables the identification of critical surrogate outcomes, including the density of tumor-infiltrating lymphocytes (TILs) and the clonal expansion of the T cell repertoire, providing high-resolution insights into the vaccine-induced remodeling of TME. Currently, several metrics are used to evaluate immunological efficacy, with post-vaccination antigen immunogenicity serving as a primary immunological endpoint. Common methods include the ELISpot assay to identify antigen-specific T cell clones, and flow cytometry using peptide-MHC conjugates (such as tetramers) to quantify antigen-specific T cells (16, 20, 21, 23, 33). Furthermore, these traditional assays are increasingly augmented by multiparametric flow cytometry and single-cell RNA sequencing, which provide unprecedented granularity in immune profiling. These advanced platforms facilitate a granular dissection of the T cell phenotypic landscape, encompassing polyfunctionality, exhaustion states, and T cell receptor clonal breadth, thereby generating multidimensional data with superior predictive value for long-term clinical outcomes in both adjuvant therapies and MRD-positive settings (96).

While T cell-mediated antitumor immunity has been extensively characterized, the roles of other immune subsets remain less defined. Emerging evidence suggests that B cell responses and the architecture of tertiary lymphoid structures (TLS) are also very important, providing a specialized niche that sustains potent and long-lasting immunosurveillance. Unlike transient T cell activation, the presence of tumor-associated B cells and plasma cells within TLS correlates with enhanced antigen presentation and the production of high-affinity antibodies, fostering a specialized niche for sustained anti-tumor immunosurveillance (97–99). Integrating comprehensive clinical and immunological assessments are key to understanding how TCVs impact patients’ outcomes and immune systems, and should be standardized across all clinical trial protocols. This data-driven approach is vital for advancing cancer treatment. With solid evidence from well-designed trials, cancer vaccines could revolutionize the therapeutic landscape, offering new hope in the fight against the disease.

## Conclusion

The induction of potent tumor-specific cytotoxic T cell responses remains the definitive hurdle in the clinical maturation of cancer vaccines. As this review has detailed, the focus of the field has shifted toward multi-modal strategies designed to elicit potent and durable immunity that translates into measurable survival benefits and enhanced quality of life. The path forward lies in the synergistic integration of TCVs with agents that neutralize immunosuppressive factors within the TME, with optimal therapeutic approaches, supported by rigorous clinical and immunological monitoring. By bridging the gap between basic immunobiology and rational clinical design, specifically through the targeting of MRD and the use of combination regimens, we are laying the groundwork for a transformative era. Ultimately, these advancements pave the way for therapeutic cancer vaccines to integrate into future clinical paradigms to improve patient outcomes.
