# Supplementary material for: Therapeutic cancer vaccines: navigating clinical translation and multimodal synergy
Source: Front Immunol. 2026 May 12;17:1818121. doi: 10.3389/fimmu.2026.1818121 (PMC13201480; doi:10.3389/fimmu.2026.1818121)
Supplement: Supplementary file 1 [file DataSheet1.docx]

**SUPPLEMENTARY APPENDIX**

**Title:** Therapeutic cancer vaccines: Navigating clinical translation and multimodal synergy

Tan-Huy Chu, MD-PhD^1^,+; Thi Thuy Linh Huynh, MSc^1^; Le-Tri Phuong, MD^2^

**Supplemental Method: Selection criteria of therapeutic cancer vaccine clinical trials in Table 1**

For this narrative review, a structured search was conducted using ClinicalTrials.gov on 25 March 2026 to identify relevant clinical trials of therapeutic cancer vaccines (TCVs). The search strategy combined condition-related terms (“Cancer” OR “Neoplasm” OR “Tumor” OR “Tumour”) with other-related terms (“dendritic cell vaccine” OR “peptide vaccine” OR “neoantigen vaccine” OR “tumor lysate vaccine” OR “whole cell vaccine” OR “mRNA cancer vaccine” OR “DNA cancer vaccine” OR “therapeutic cancer vaccine” OR “cancer vaccine”). Filters were applied to include only interventional studies that were completed, had results, and were completed from 2009 onward, yielding 122 results.

After downloading the records, studies were screened and cross-checked against corresponding publications in PubMed to ensure availability of peer-reviewed data. A total of 42 studies had corresponding publications and were selected for further evaluation.

Abstracts and, where necessary, full texts were reviewed to verify study design, methodology, and reported outcomes. The selection process was conducted independently by two reviewers, with iterative discussion to resolve discrepancies and ensure consistency. Studies were excluded at this stage if they (i) did not evaluate therapeutic cancer vaccines, (ii) lacked sufficient clinical outcome data, (iii) were not primary clinical trial reports (e.g., reviews or secondary analyses), or (iv) had incomplete or unclear reporting of study methodology or results.

Ultimately, 32 trials met the inclusion criteria and were included in the overview table of completed therapeutic cancer vaccine clinical trials (2009–present) (**Table 1**).


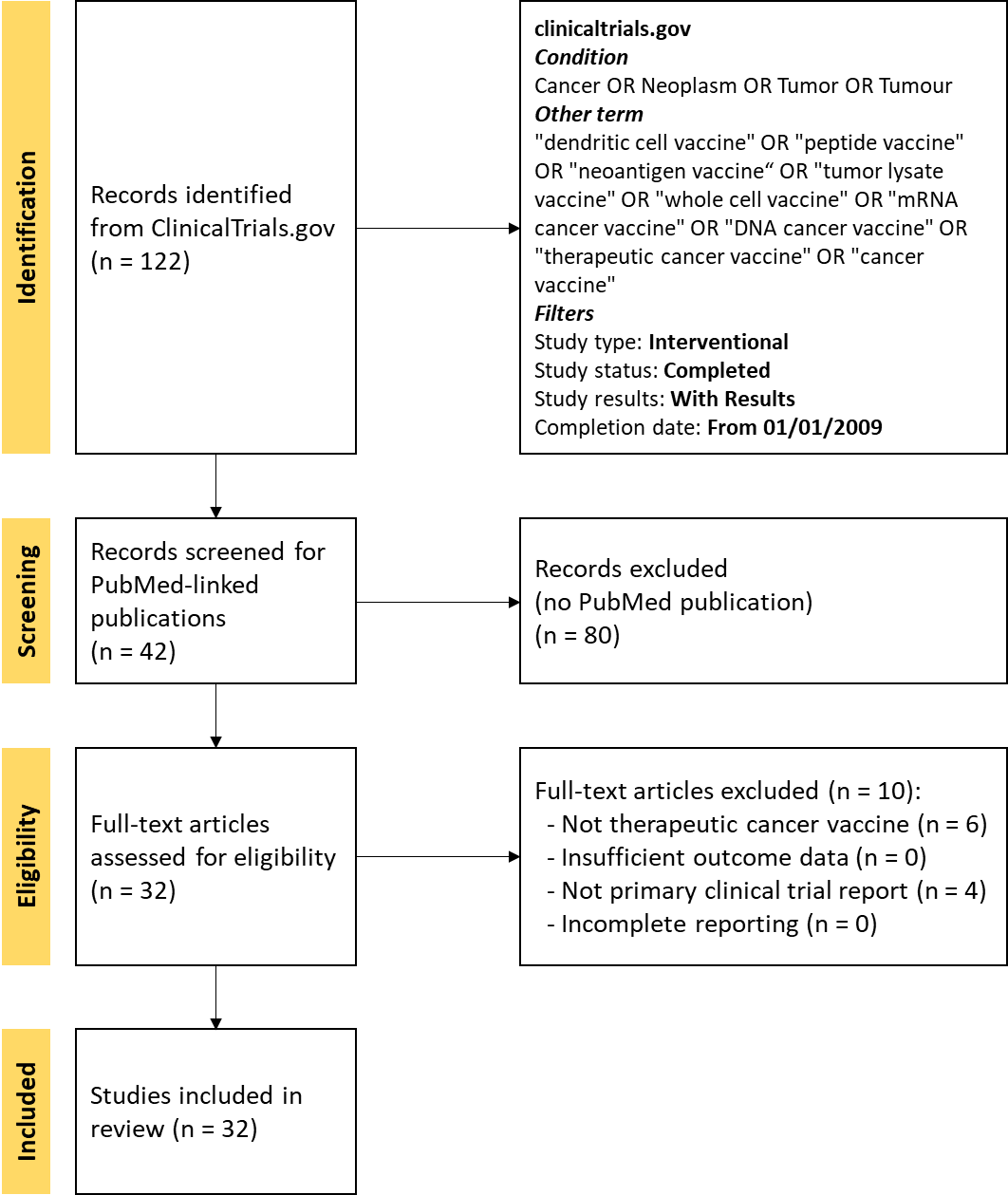


**Supplemental Figure 1. Study selection flow diagram.**
A total of 122 clinical trials were identified through ClinicalTrials.gov. After screening for the availability of corresponding publications in PubMed, 42 studies were eligible for full-text assessment. Following abstract and full-text review, 10 studies were excluded due to a lack of relevant data. Ultimately, 32 trials were included in the final qualitative synthesis.
